# The reducing effect of visuospatial cognitive load on alcohol craving in problematic drinkers

**DOI:** 10.3389/fpsyg.2026.1923789

**Published:** 2026-09-03

**Authors:** Mi-Young Park, InGyeong Kim, MinJae Kim, Gi-Eun Lee, Jang-Han Lee

**Affiliations:** Department of Psychology, Chung-Ang University, Seoul, Republic of Korea

**Keywords:** alcohol craving, attentional bias, cognitive load, elaborated intrusion theory, eye-tracking, problematic drinking, visuospatial working memory

## Abstract

**Introduction:**

Alcohol use disorder is a significant global health concern, and craving serves as a central mechanism sustaining problematic drinking and a critical, cognitively accessible intervention target. This study examined whether visuospatial working memory depletion could reduce alcohol craving and attentional bias in problematic drinkers by disrupting the elaboration of intrusive alcohol-related thoughts.

**Methods:**

Following screening with the Alcohol Use Disorders Identification Test (AUDIT; score ≥ 12), 50 problematic drinkers were randomized to a Load or No Load Group. The Load Group completed the Corsi block-tapping task and the No Load Group a matched no-load control task. Self-reported craving and eye-tracking indices of attentional bias were assessed before and after the intervention. Early attentional orienting was indexed by time to first fixation and late attentional maintenance by total fixation duration to simple and complex alcohol stimuli. Five participants were subsequently excluded for poor eye-tracking data quality. The final sample comprised 45 (Load Group n = 23, No Load Group n = 22).

**Results:**

The Load Group showed significantly reduced Alcohol Urge Questionnaire (AUQ) scores post-task. While no significant differences emerged in early attentional bias, the Load Group showed suppressed late attentional bias toward alcohol cues, whereas the No Load Group showed a significant increase.

**Discussion:**

This indicates that visuospatial cognitive load blocked the progressive strengthening of attention toward alcohol cues. The load manipulation selectively suppressed late, top-down attentional maintenance, the component most closely linked to craving and relapse risk, while leaving automatic orienting intact. This pattern therefore suggests that the load manipulation acts on the elaboration stage through which alcohol cues develop into sustained urge. These findings suggest that visuospatial cognitive load may function as a brief, non-invasive, and self-administered craving intervention that could serve as an accessible adjunct to, rather than a substitute for, formal treatment, with potential relevance to other addictive behaviors characterized by heightened cue salience.

## 1 Introduction

Alcohol use disorder (AUD) constitutes a major global public health burden, affecting approximately 400 million people and accounting for 2.6 million deaths annually (World Health Organization, 2024). South Korea presents a distinctively high-risk profile, with a lifetime AUD prevalence of 11.6%, which is the highest among all mental disorders assessed (Rim et al., 2023), and a monthly binge drinking rate of 47.9% in men and 26.3% in women (Kim, 2025). Yet only 2.6% of affected individuals utilize mental health services, pointing to a critical gap in treatment access (Lee et al., 2025). Problematic drinkers report elevated craving levels, and episodes of craving are accompanied by vivid, multisensory imagery of alcohol use that is more sensorily rich than in light drinkers (Kavanagh et al., 2009, 2013). This heightened vividness directly amplifies craving intensity and associated behavioral consequences, making the disruption of this elaborative process a central therapeutic target (Riley et al., 2018; Vinci et al., 2016).

Traditional incentive-sensitization models frame craving as an automatic, dopaminergically driven wanting response, in which repeated alcohol exposure sensitizes neural reward circuits so that alcohol cues trigger motivational pull regardless of explicit liking (Robinson and Berridge, 1993). Although this account explains the automaticity of cue reactivity, it conceptualizes craving as a deeply ingrained neural disposition and leaves limited room for immediate cognitive intervention. Critically, the incentive-sensitization model does not specify what occurs between cue detection and the full subjective experience of craving. Without such a cognitive stage, immediate intervention would have no clear point of entry. Tiffany's cognitive processing model constitutes a further explanation of craving that likewise departs from a purely automatic view of drug-use behavior. It proposes that repeated drug use becomes governed by automatized action schemata, whereas craving reflects effortful, non-automatic processing recruited when an activated drug-use sequence is interrupted or inhibited, or when additional processing is required to facilitate its completion (Tiffany, 1990; Tiffany and Conklin, 2000). Similar to Elaborated Intrusion (EI) theory, this model posits a resource-dependent intermediate cognitive stage following cue-triggered activation and can therefore also account for the effects of general cognitive load. However, without a modality-specific imagery mechanism or a distinct role for visuospatial working memory (VSWM), the model leaves unclear why visuospatial interference in particular, rather than cognitive load in general, should attenuate craving.

The EI theory offers a complementary and cognitively accessible account, in which transient associative intrusions become craving only when attended to and elaborated within working memory (Kavanagh et al., 2005; May et al., 2015). These intrusions originate as brief, automatic associations triggered by alcohol-related cues or internal states. When such intrusions capture attention, working memory resources are recruited to elaborate them into vivid sensory imagery mentally simulating the taste, smell, or social context of drinking. In problematic drinkers, repeated alcohol use builds a rich store of alcohol-related memories and sensory information, and intrusive thoughts automatically recruit this material, elaborating mental imagery into increasingly vivid and concrete representations. It is this elaboration process, rather than the initial intrusion itself, that generates and sustains the subjective experience of craving (Kavanagh et al., 2009). This elaboration stage is amenable to intervention because it depends on limited working memory capacity (Kavanagh et al., 2005; May et al., 2015). As alcohol craving is sustained primarily through such sensory imagery rather than through verbal rehearsal, it is the visuospatial rather than the phonological subcomponent of working memory that is most centrally recruited in this elaboration process (Baddeley and Andrade, 2000). VSWM stores and regulates the vividness of mental imagery, making it the cognitive subsystem most directly implicated in craving.

Neurophysiological evidence converges with these behavioral findings. Combined fMRI–EEG investigations of alcohol craving reveal coordinated activity across the dorsal and pregenual anterior cingulate cortex, nucleus accumbens, posterior cingulate cortex, amygdala, and parahippocampal gyrus, a network that detects the motivational conflict between wanting and restraint and generates anticipation of reward through memories of past drinking experiences. This co-activation is accompanied by increased beta-band power, indexing heightened cortical engagement, and theta-band hyperconnectivity between these regions. This pattern is interpreted as reflecting a unified central craving network rather than a set of isolated neural events (Huang et al., 2018). Consistent with the theory's emphasis on modality-specific imagery, reviews of the cue-reactivity literature further indicate that the neural circuitry engaged by alcohol cues differs according to sensory modality, with olfactory and gustatory cues recruiting mesocorticolimbic reward regions such as the nucleus accumbens and ventral tegmental area (Goodyear, 2019).

Evidence from food-craving research indicates that performing a concurrent cognitive task can attenuate craving (Van Dillen and Andrade, 2016). This finding is theoretically relevant to alcohol craving because EI theory proposes that cravings across appetitive targets are sustained by a shared process of sensory-imagery elaboration within limited-capacity working memory (Kavanagh et al., 2005; May et al., 2015). Prior research suggests that cognitive-load tasks may attenuate craving and related appetitive responses to food (Andrade et al., 2012), alcohol (Sharbanee et al., 2014), and other substances (Skorka-Brown et al., 2014). High VSWM load administered before alcohol-cue retrieval has been shown to reduce subsequent retrieval-induced desire in problematic drinkers, indicating that the effects of prior VSWM engagement can persist beyond the loading period itself and continue to influence subsequent craving-related processing (Kaag et al., 2018). This pattern does not establish that VSWM remains continuously occupied after task completion, but it supports the possibility of a short-term post-task effect of prior VSWM engagement. The Corsi block-tapping task (CBTT) is particularly well-suited for this purpose, as it requires the sequential encoding and reproduction of visuospatial patterns, placing direct demand on the same VSWM resources implicated in imagery elaboration (Corsi, 1972; Kessels et al., 2000). Unlike tasks that engage verbal rehearsal or general attentional processes, it selectively targets the visuospatial subsystem, consistent with the interference account of VSWM-based interventions (May et al., 2015). Accordingly, the CBTT is expected to impose sustained demands on VSWM during task performance. This sustained demand may reduce the resources available for constructing and maintaining alcohol-related imagery, which could in turn disrupt the elaboration of intrusive alcohol-related thoughts into craving (Kavanagh et al., 2009; Kaag et al., 2018).

However, these effects have been demonstrated either in the absence of external alcohol cues or in response to single, standardized images presented under controlled laboratory conditions, leaving it unclear whether visuospatial cognitive load similarly attenuates engagement with the more complex, socially embedded cues that characterize real-world drinking contexts. Indeed, the extent to which visuospatial elaboration is recruited depends on the complexity of the alcohol cue itself. Unlike a solitary beverage image, a complex scene embeds alcohol within the presence of other people, familiar settings, and shared activities that closely resemble the circumstances of actual past drinking episodes (Miller and Fillmore, 2010). Simple alcohol images carry perceptual salience sufficient to capture attention in a bottom-up, stimulus-driven manner, whereas such socially and contextually embedded complex scenes embed alcohol within the features that typically accompany drinking episodes, thereby activating a richer network of episodic memory associations and demanding greater top-down elaborative engagement (Creswell, 2021; Waddell et al., 2026). This contextual similarity is expected to more readily reactivate specific episodic drinking memories embedded in their original social context, which must then be reconstructed into vivid sensory imagery through top-down elaboration (Kavanagh et al., 2009). Complex scenes are consequently rendered more susceptible to the interference effects of visuospatial cognitive load than simple stimuli.

Complex alcohol scenes are expected to recruit greater elaborative processing, which should be reflected not only in subjective craving (Kavanagh et al., 2005) but also in the temporal allocation of attention, particularly in sustained attentional maintenance rather than initial orienting (Field et al., 2004). Attentional bias toward substance-related cues reflects an imbalance between an overactivated impulsive system and reduced executive and working memory control (Bollen et al., 2022; Wiers et al., 2007). This cue-driven capture of attention can be viewed as a behavioral expression of the salience attribution that characterizes addictive disorders (Zilverstand et al., 2018). Early orienting is driven by bottom-up, stimulus-driven processes that automatically draw attention to such cues, and is therefore relatively resistant to working memory load (Field et al., 2004). In contrast, late attentional maintenance is governed by top-down, goal-directed processes that depend on working memory resources, rendering it selectively vulnerable to visuospatial cognitive load (Field et al., 2004; Judah et al., 2013; Van der Stigchel and Hollingworth, 2018). Prior work in heavy and problematic drinkers reveals a consistent pattern in which initial orienting toward alcohol cues indexed by time to first fixation is comparable to that of light drinkers (Field et al., 2004). However, problematic drinkers reliably show prolonged attentional maintenance toward alcohol cues, indexed by longer total fixation duration, and this late component, but not initial orienting, correlates with self-reported craving, consumption, and relapse risk (Bollen et al., 2022; Field et al., 2004; Manchery et al., 2017).

Given that VSWM constitutes a fundamental component of the eye movement system (Van der Stigchel and Hollingworth, 2018), its depletion would be expected to selectively attenuate this late attentional maintenance rather than initial orienting, but this has yet to be directly tested. Directly confirming that craving is more closely linked to attentional maintenance than to initial orienting (Field et al., 2004) requires measuring both stages, which is in turn essential for identifying at which stage of attentional bias VSWM depletion selectively intervenes. If early orienting remains intact while late attentional maintenance is selectively reduced, this pattern would indicate that VSWM depletion disrupts elaboration specifically, rather than attention to alcohol cues more generally. To examine this possibility in the present study, both simple and complex alcohol stimuli were included based on the theoretical distinction between bottom-up and top-down elaborative engagement, allowing these differential elaborative processes to be dissociated.

Prior research has often relied on no-load baselines without controls (Adams et al., 2019), and direct verification of VSWM load effects in problematic drinkers remains limited (Kaag et al., 2018). The present study investigated the causal effect of visuospatial cognitive load on alcohol craving and attentional bias in problematic drinkers using a pre-post design, comparing the Load Group with the No Load Group. To isolate this effect, several factors known to influence craving and attentional bias were assessed and accounted for. A large-scale study of over 20,000 adults across three Eastern European countries found that problematic drinking nearly doubled the risk of depressive symptoms regardless of country or sex, consistent with the well-documented comorbidity between the two conditions (Bell et al., 2014). Anxiety and stress likewise exacerbate cue reactivity in alcohol-dependent individuals, and the attentional bias arising under such conditions is closely tied to physiological stress regulation (Garland et al., 2012). Similarly, the more positively an individual anticipates the consequences of drinking, the more strongly alcohol cues capture attention and motivational engagement, which in turn intensifies the subjective experience of craving in response to those cues (Field and Cox, 2008). Depression, state anxiety, and outcome expectancies were therefore measured. Accordingly, three hypotheses were formulated. It was predicted that the Load Group would show a greater reduction in self-reported alcohol craving than the No Load Group (H1). It was predicted that early attentional orienting, indexed by time to first fixation, would not differ between groups (H2). It was predicted that late attentional maintenance, indexed by total fixation duration, would be selectively attenuated in the Load Group, with this suppression expected to be more pronounced for complex than for simple alcohol images (H3).

## 2 Materials and methods

### 2.1 Participants

Participants were recruited via an online bulletin board at Chung-Ang University, Seoul, South Korea. As a screening procedure, 90 adults who reported problems with their drinking patterns completed the Korean version of the Alcohol Use Disorders Identification Test (AUDIT; Saunders et al., 1993). Based on the cut-off point for problematic drinkers in Korea, an AUDIT score of 12 or higher was used as the inclusion criterion (Lee et al., 2000), and 50 of the screened adults met this threshold and were randomly assigned to the Load or No Load Group. To control for the potential influence of comorbid conditions, depressive mood and anxiety were assessed using the Beck Depression Inventory (BDI) and State–Trait Anxiety Inventory (STAI). Five participants were subsequently excluded due to poor eye-tracking data quality, defined a priori as an on-task recording percentage or alcohol-related attentional pattern exceeding 1.5 SD from the overall mean. The final sample comprised 45 participants, with 23 in the Load Group (6 men, 17 women) and 22 in the No Load Group (6 men, 16 women). The study protocol was reviewed and approved by the Institutional Review Board of Chung-Ang University (IRB No. 1041078-201911-HR-352-01).

### 2.2 Procedure

The study comprised three phases: baseline, intervention, and post-intervention. Upon arrival at the laboratory, participants received a brief explanation of the procedure and their rights as research participants, and provided written informed consent. Participants completed the baseline questionnaires, namely the BDI, STAI-State (STAI-S), STAI-Trait (STAI-T), Alcohol Expectancy Scale (AES), Alcohol Urge Questionnaire (AUQ), and Alcohol Craving Visual Analog Scale (VAS), followed by the pre-intervention free-viewing task. The Load Group then completed the 4-min CBTT, while the No Load Group completed a matched no-load control task. Finally, participants completed the post-intervention assessments in the fixed order of STAI-S, AUQ, VAS, and the second free-viewing task. The AES was administered both to assess outcome expectancies as a control variable and to activate alcohol-related cognitions prior to the free-viewing task. Each participant was seated approximately 68–70 cm from the monitor and instructed to view the screen freely, as if watching a movie or television, while keeping their body still. The No Load Group received the same instructions as the Load Group but were additionally informed that the laboratory program occasionally malfunctions and that they might be required to wait before the task could continue (Van Dillen et al., 2012). The program was designed not to load the task and to terminate with a static “Load Error” message (Skorka-Brown et al., 2014); after the message disappeared, participants were informed that the computer task was no longer required. This control condition was matched to the Load condition in duration and screen engagement while imposing no working-memory demand. They were then debriefed regarding the original purpose of the experiment and compensated with 8,000 won. To prevent the transmission of task-related information to subsequent participants, all participants were individually instructed not to disclose any details of the experiment.

### 2.3 Self-report measures

#### 2.3.1 The alcohol use disorders identification test (AUDIT)

The Alcohol Use Disorders Identification Test (AUDIT; Saunders et al., 1993) is a 10-item scale developed to assess hazardous alcohol intake, with items capturing the quantity and frequency of drinking, symptoms of alcohol dependence and tolerance, and alcohol-related negative consequences. Each item is scored from 0 to 4, with higher total scores indicating more harmful drinking patterns (Lee et al., 2000). In the present study it was used solely for categorical screening of problematic drinkers (original α = 0.93, 0.81, 0.65 across subdomains; present α = 0.64); as the scale served a screening function only, its low internal consistency does not compromise the primary findings.

#### 2.3.2 Alcohol urge questionnaire (AUQ)

The Alcohol Urge Questionnaire (AUQ; Bohn et al., 1995; Kim et al., 2008) is an 8-item self-report measure of current alcohol craving that assesses respondents' momentary thoughts and feelings regarding alcohol consumption, and is widely used in both clinical and experimental craving research (Drummond and Phillips, 2002). Items are rated on a seven-point Likert scale ranging from “No” (0) to “Yes” (6) with reference to the present moment. It was administered to assess pre- and post-task craving (original α = 0.91; present α = 0.79).

#### 2.3.3 Alcohol craving visual analog scale (VAS)

The Alcohol Craving Visual Analog Scale (VAS; Choi et al., 2002) is a single-item scale assessing the momentary desire to drink, ranging from “Not want to drink” (0) to “Very strongly want to drink” (100). It was administered before and after the task as a complementary index of self-reported craving.

#### 2.3.4 Alcohol expectancy scale (AES)

The Alcohol Expectancy Scale (AES; Kim, 2000; Leigh and Stacy, 1993) is a 34-item, six-point Likert scale assessing positive and negative outcome expectancies regarding alcohol. It comprises four positive expectancy factors (social facilitation, sex, fun, and negative reinforcement) and four negative expectancy factors (negative emotions, negative social consequences, physical effects, and cognitive-performance impairment). Because alcohol expectancies influence both attentional bias toward alcohol cues and subjective craving (Field and Cox, 2008), the AES was included to control for these expectancy effects (original α = 0.94 positive, 0.88 negative; present α = 0.85).

#### 2.3.5 The Beck Depression Inventory (BDI)

The Beck Depression Inventory (BDI; Beck et al., 1988; Lee et al., 1995) is a 21-item, four-point Likert scale assessing the presence and severity of depressive symptoms across emotional, cognitive, motivational, and somatic domains over the preceding 2 weeks, with respondents selecting the option that best reflects their current state. It was included given the well-documented comorbidity between depression and problematic drinking (Bell et al., 2014; original mean α = 0.86 psychiatric, 0.81 non-psychiatric; present α = 0.92).

#### 2.3.6 State-trait anxiety inventory (STAI)

The State-Trait Anxiety Inventory (STAI; Kim and Shin, 1978; Spielberger et al., 1983) is a 20-item measure of state and trait anxiety rated on a four-point Likert scale, ranging from “Not at all” (1) to “Very much so” (4) for the state version (STAI-S) and from “Almost never” (1) to “Almost always” (4) for the trait version (STAI-T). Post-task STAI-S served as a covariate to control for the known influence of anxiety on alcohol-seeking behavior and ego-depletion (Garland et al., 2012; Lewandowski et al., 2012; original α = 0.86–0.95; present α = 0.95 STAI-S, 0.93 STAI-T).

### 2.4 Behavioral tasks

#### 2.4.1 The Corsi block-tapping task (CBTT)

**Figure 1:**
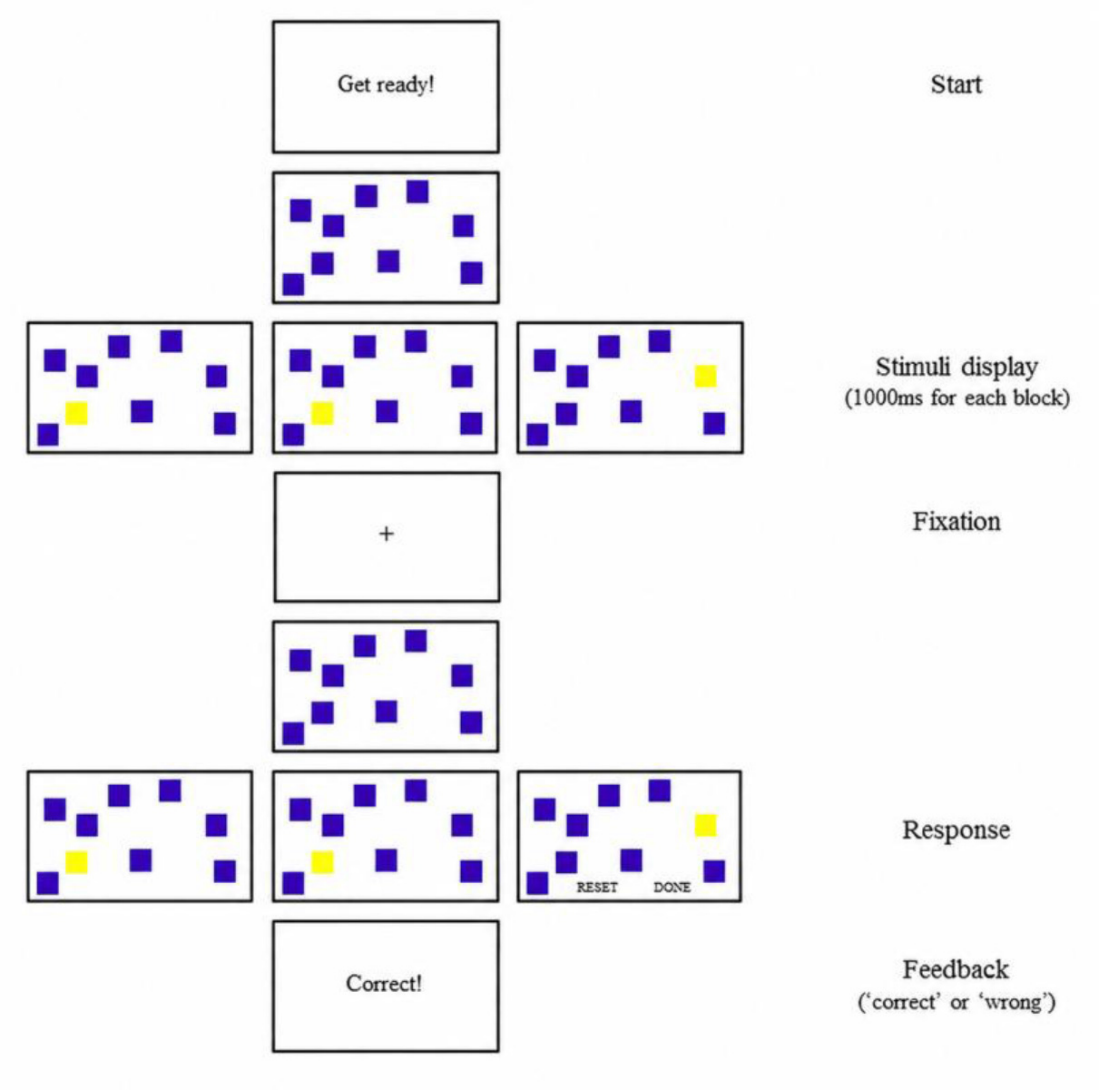
Procedure for the Corsi block-tapping task.

The digital Corsi Block-Tapping Task (CBTT; Corsi, 1972; Kessels et al., 2000) was administered to induce VSWM load. The CBTT is a well-established measure of visuospatial span that requires the temporary storage and sequential reproduction of spatial information, thereby selectively taxing the visuospatial sketchpad component of working memory (Baddeley and Logie, 1999). Its use in the present study was premised on evidence that VSWM load disrupts the imagery elaboration process central to craving, as posited by Elaborated Intrusion theory (Kavanagh et al., 2005; see Figure 1).

The task was presented on a black background screen displaying nine blue blocks (30 × 30 mm). Each trial began with a “Get ready!” prompt, after which a subset of blocks was illuminated in yellow sequentially, each for 1,000 ms. The inter-stimulus interval between the offset of one block and the onset of the next was 450 ms. Participants were instructed to observe and encode the sequential order of the illuminated blocks. Following the stimulus sequence, a fixation cross was briefly presented to minimize inter-trial interference, after which participants entered the response phase by clicking the corresponding blocks in the correct order using a computer mouse. A “Reset” button allowed participants to clear and re-enter their response, and clicking “Done” confirmed the final response. Corrective feedback was provided after each trial in the form of a “Correct!” or “Wrong” message.

Sequence length began at two blocks and increased by one block after every two trials at each span level, up to a maximum of nine blocks. The task terminated automatically if the participant responded incorrectly on both trials at a given span level. Performance was indexed by the longest correctly reproduced sequence (block span) and the total number of correct trials (total score).

#### 2.4.2 The free-viewing task

**Figure 2:**
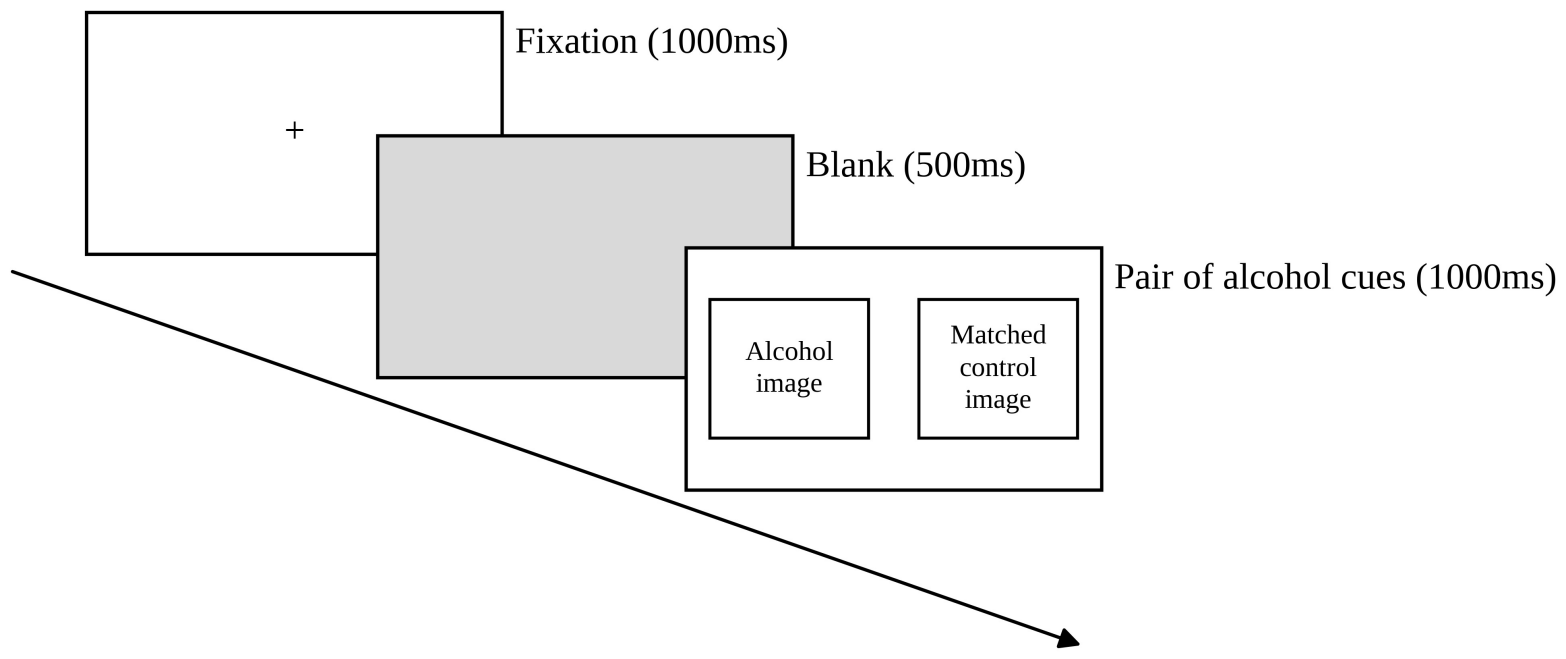
Procedure for the free-viewing task.

To assess changes in attentional bias toward alcohol, a free-viewing task was administered before and after the intervention (see Figure 2). Each trial began with a 1,000-ms central fixation cross, followed by a 500-ms blank screen and then a 1,000-ms presentation of a paired alcohol stimulus and a content-matched non-alcohol control displayed side by side. Participants were instructed to view the screen freely, as if watching television or a movie, while keeping their body still. The alcohol and control images were obtained through an online image search. Thirty alcohol-related images were each paired with a non-alcohol control image on the basis of scene content. For the 20 simple pairs, both images depicted a solitary beverage. For the 10 complex pairs, the images depicted a comparable action or social context, with the alcoholic beverage replaced by a non-alcoholic beverage (e.g., a hand holding a glass of beer paired with a hand holding a glass of cola). Thus, content matching referred to qualitative matching of the beverage format, depicted action, or social context, rather than formal equivalence in low-level visual characteristics. An independent sample of ten undergraduates who did not participate in the main experiment rated each image for its relevance to alcohol (1 = not at all related; 7 = very related), valence (1 = negative; 7 = positive), and arousal (1 = sedated; 7 = aroused) on 7-point Likert scales. These ratings were obtained to assess whether the images were consistent with their intended alcohol-related or non-alcohol categories and to characterize their affective properties. In the free-viewing task, 20 neutral–neutral image pairs unrelated to drinking, drawn from the Food-pics database (Blechert et al., 2014), were interspersed among the alcohol–control target pairs as filler trials. These trials were included to reduce the predictability of alcohol-cue presentation and thereby limit deliberate gaze strategies, such as intentionally directing attention toward or away from alcohol-related images. The location (left vs. right) of the alcohol-related picture was counterbalanced across trials to control for a leftward viewing bias.

### 2.5 Apparatus

Participants' eye movements were tracked using an eye tracker (iView X™ Red-IV Eye Tracking System, SensoMotoric Instruments GmbH, Berlin, Germany). Participants were seated 68–70 cm in front of a 23-inch wide screen. The eye tracker was interfaced with a desktop computer that recorded the data. Viewing was binocular, although eye movements were recorded from each participant's right eye at a sampling rate of 60 Hz. A fixation was registered when eye movements remained stable for at least 60 ms. To measure attentional processing of alcohol stimuli, two areas of interest (AOIs) were defined for each trial, corresponding to the total area of the alcohol stimulus and that of the matched non-alcohol stimulus. For the cognitive load task, the CBTT was presented using Inquisit software version 5.0.14.0 (Millisecond Software, 2018) on a 17-inch laptop, which recorded each participant's number of correct responses and total score.

### 2.6 Data analysis

A sensitivity power analysis was conducted using G^*^Power 3.1.9.7 (Faul et al., 2009). For the primary within-subject Group × Time interaction (2 × 2 repeated-measures design; *N* = 45, α = 0.05, assumed correlation among repeated measures = 0.5), the design afforded 80% power to detect an effect size of f = 0.21 (ηp^2^ ≈ 0.044). Baseline group equivalence in age, depression, state and trait anxiety, positive and negative alcohol expectancies, and sex ratio was evaluated using independent-samples *t*-tests and chi-square tests. Filler trials were excluded from the attentional bias analyses. To test the three hypotheses, separate 2 (Group: Load vs. No Load) × 2 (Time: pre-task vs. post-task) repeated-measures analyses of covariance (ANCOVAs) were conducted for the corresponding outcome measures. H1 was tested using the AUQ and VAS as measures of self-reported alcohol craving. H2 was tested using early attentional orienting, indexed by time to first fixation. H3 was tested using late attentional maintenance, indexed by total fixation duration. Post-task STAI-S was included as a covariate because it showed a between-group difference and because state anxiety may influence alcohol-seeking behavior and ego-depletion-related processes (Garland et al., 2012; Lewandowski et al., 2012). Because the AUQ and VAS both tested H1, a Holm-Bonferroni correction was applied to these two Group × Time interaction tests to control for multiple comparisons. The two indices used to evaluate attentional bias were time to first fixation as a measure of early attentional processing and total fixation duration as a measure of late attentional processing (Field et al., 2004). Time to first fixation was defined as the latency in seconds from stimulus onset to the beginning of the first fixation on the alcohol stimulus, reflecting initial orienting of attention. Total fixation duration was defined as the cumulative time spent fixating on the alcohol stimulus across the entire trial, reflecting the maintenance of attention (Field et al., 2004). Simple and complex stimuli were analyzed using separate 2 (Group) × 2 (Time) repeated-measures ANCOVAs for each stimulus type rather than as a factor within a single model. As a result, these analyses do not provide a formal statistical test of whether the effect of cognitive load differed between simple and complex stimuli, which would require a three-way Group × Time × Stimulus Type interaction (Nieuwenhuis et al., 2011). Stimulus type was not incorporated into the analyses of self-reported craving, measured by the AUQ and VAS, because these measures were administered as single global ratings rather than in response to individual stimuli and therefore could not be decomposed by stimulus type. Accordingly, the primary Group × Time analyses for H1, H2, and H3 were hypothesis-driven and directly tested via the ANCOVAs described above, whereas the apparent differences between simple and complex stimuli and the proposed elaboration-based mechanism linking late attentional maintenance to craving reduction were not formally tested and should be regarded as tentative interpretations rather than confirmed findings. Significant two-way interactions were decomposed using post-hoc paired-samples *t*-tests within each group. Given the focused a priori comparisons, no further multiple-comparison correction was applied. All statistical analyses were performed using SPSS 25.0 for Windows.

## 3 Results

### 3.1 Demographic characteristics

To verify group equivalence at baseline, demographic and psychological characteristics of the Load and No Load Groups were compared using independent-samples t-tests and chi-square tests. No significant differences emerged in age [*t* (43) = −0.865, *p* = 0.392], BDI [*t* (43) = 1.156, *p* = 0.254], trait anxiety [*t* (43) = 0.690, *p* = 0.494], pre-task state anxiety [*t* (43) = 1.310, *p* = 0.197], AES-Positive [*t* (43) = −0.415, *p* = 0.680], or sex ratio [χ^2^(1) = 0.008, n.s.]. A nominal between-group difference was observed in AES-Negative [*t* (43) = −2.024, *p* = 0.049], with lower scores in the Load Group (*M* = 25.35) than in the No Load Group (*M* = 31.55). A significant group difference was also found in post-task STAI-S scores, with the Load Group reporting higher scores than the No Load Group [*t* (43) = 2.570, *p* < 0.05, Cohen's *d* = 0.769]. Post-task STAI-S was therefore included as a covariate based on the significant between-group difference and evidence linking stress-related affective states to alcohol cue-reactivity and attentional bias (Garland et al., 2012; see Table 1).

**Table 1:** Mean (SD) for demographic characteristics.

Variable	Load group (*n* = 23)	No load group (*n* = 22)	*t*	*d*
Age (years)	22.609 (2.759)	23.367 (3.094)	−0.87	−0.26
Sex (male/female)	6/17	6/16	0.01^a^	0.01^b^
BDI	12.652 (10.849)	9.546 (6.566)	1.16	0.35
STAI-S before	47.044 (12.797)	42.409 (10.780)	1.31	0.39
STAI-S after	47.565 (11.236)	40.000 (8.200)	2.57^*^	0.77
STAI-T	49.217 (12.247)	46.812 (11.027)	0.69	0.21
AES-P	81.130 (8.108)	82.273 (9.920)	−0.42	−0.12
AES-N	25.348 (7.402)	31.546 (12.592)	−2.02^*^	−0.60

Age, years; BDI, Beck Depression Inventory; STAI-S, state-trait anxiety inventory-state version; STAI-T, state-trait anxiety inventory-trait version; AES-P, alcohol expectancy scale-positive; AES-N, alcohol expectancy scale-negative. *d* = Cohen's *d*. ^a^χ^2^ value for Pearson's chi-square test. ^b^effect size for chi-square test is reported as Phi (ϕ). ^*^p < 0.05

### 3.2 Alcohol craving after intervention

#### 3.2.1 Self-reported craving

**Figure 3:**
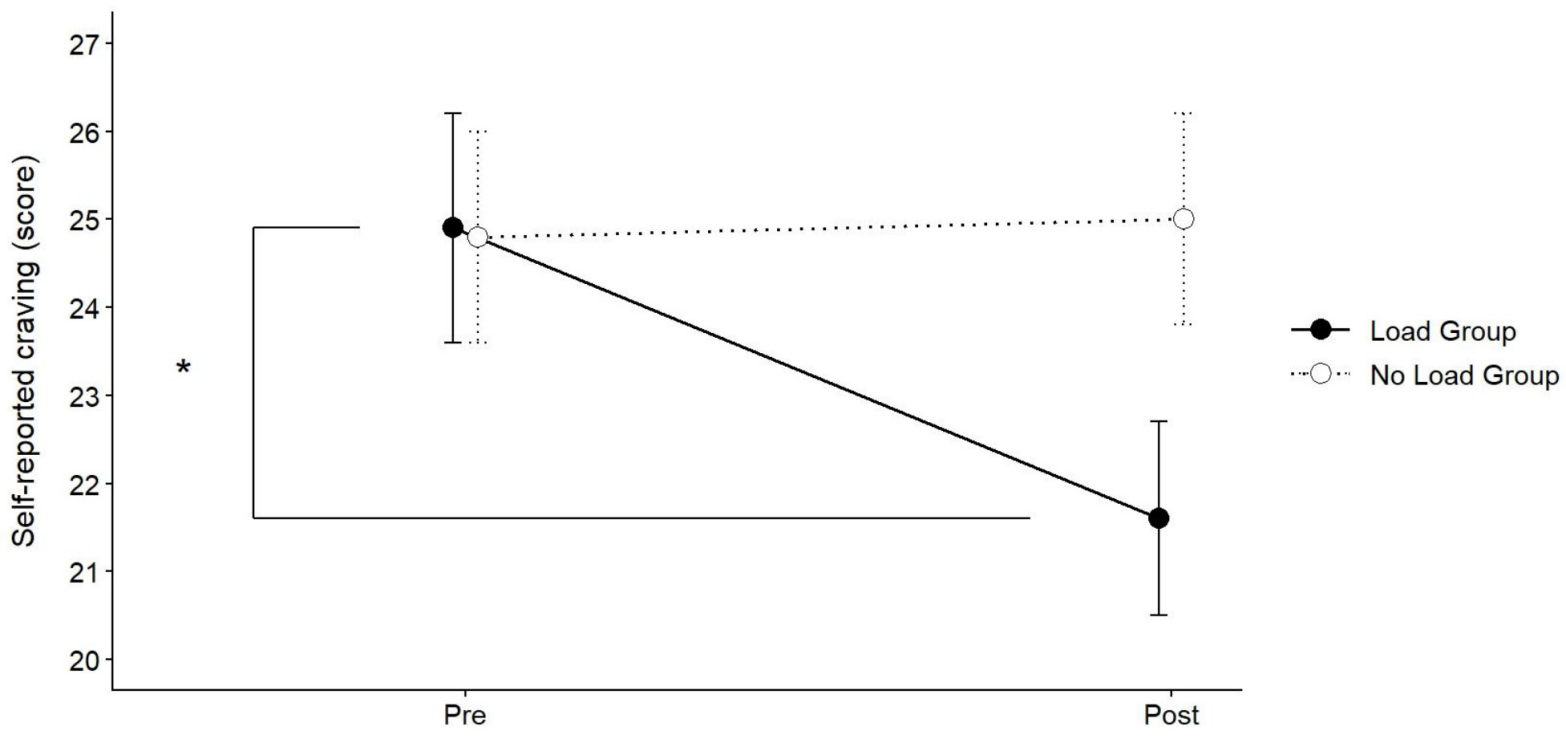
Pre- and post-intervention alcohol urge questionnaire scores by group. Error bars represent standard deviation of the mean. Pre = pre-task; Post = post-task. *p < 0.05.

To examine the influence of the cognitive load task on self-reported alcohol craving, a 2 (Group) × 2 (Time) repeated-measures ANCOVA was conducted for both the AUQ and the VAS, with a Holm-Bonferroni correction applied across these two tests to control for multiple comparisons within H1. For the AUQ, a significant Group × Time interaction emerged [*F*_(1, 42)_ = 5.406, η_p_^2^ = 0.114, *p* = 0.025, *p*_Holm_ = 0.0499]. Post-hoc comparisons revealed a significant decrease in the Load Group [Cohen's *d* = 0.407, *t* (22) = 3.324, *p* < 0.05], whereas the No Load Group showed no significant change [*t*(21) = −0.154, n.s.]. There was also a significant main effect of Group [*F*_(1, 42)_ = 7.401, η_p_^2^ = 0.150, *p* < 0.01], but no main effect of Time [*F*_(1, 42)_ = 0.130, n.s.], indicating that the cognitive load task significantly reduced perceived alcohol craving (see Figure 3). This Group × Time interaction, reflecting a craving reduction specific to the Load Group, was consistent with H1, and the associated effect size (η_p_^2^ = 0.114) corresponds to a medium effect. For the VAS, no significant Group × Time interaction was observed [*F*_(1, 42)_ = 0.308, *p* = 0.582, *p*_Holm_ = 0.582], nor were there significant main effects, indicating that craving measured by VAS was not affected by the task or time.

#### 3.2.2 Early attention process

A 2 (Group) × 2 (Time) repeated-measures ANCOVA on time to first fixation yielded no significant Group × Time interaction for simple [*F*_(1, 42)_ = 0.752, n.s.] or complex [*F*_(1, 42)_ = 0.310, n.s.] alcohol stimuli, and no significant main effects (all *ps* >0.05). These results indicate that early attentional processes were not influenced by cognitive load, time or group. As predicted, early orienting toward alcohol cues was unaffected by group, time, or cognitive load, indicating that the load manipulation did not alter the automatic, stimulus-driven capture of attention by alcohol cues.

#### 3.2.3 Late attention process

**Figure 4:**
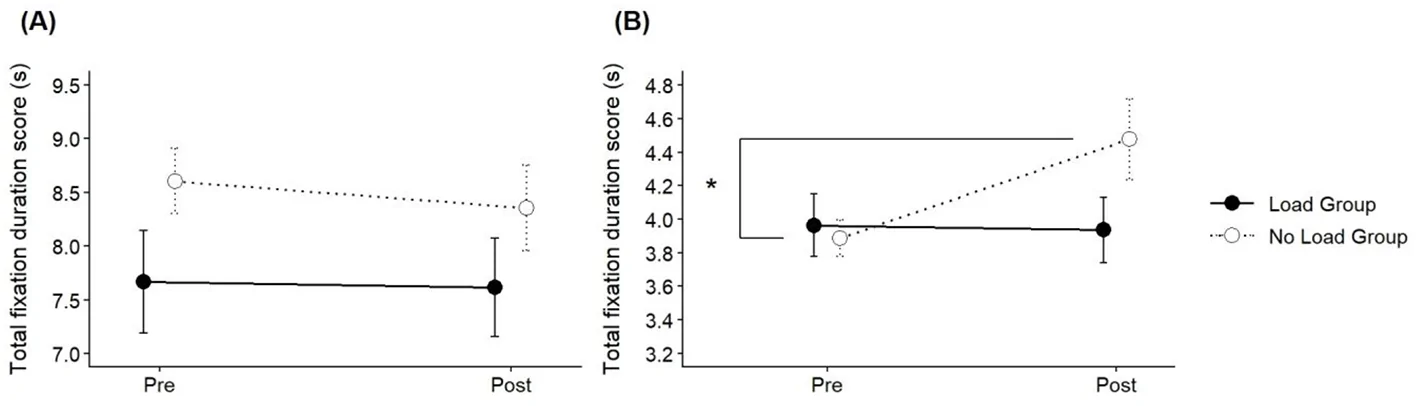
Effects of visuospatial cognitive load on late attentional bias to alcohol stimuli. **(A)** Total fixation duration to simple alcohol stimuli. **(B)** Total fixation duration to complex alcohol stimuli. Error bars represent the standard deviation of the mean. Pre = pre-task; Post = post-task. *p < 0.05.

A 2 × 2 ANCOVA on total fixation duration showed no significant interaction for simple alcohol stimuli [*F*_(1, 42)_ = 0.547, n.s.] and no significant main effects, indicating that cognitive load did not influence late attention toward simple stimuli (see Table 2). For complex alcohol stimuli, however, a significant Group × Time interaction emerged [*F*_(1, 42)_ = 4.290, η_p_^2^ = 0.093, *p* < 0.05]. Post-hoc comparisons revealed that total fixation duration significantly increased in the No Load Group from pre- to post-task [Cohen's *d* = 0.675, *t* (21) = −2.282, *p* < 0.05], whereas the Load Group showed no significant change [*t*(22) = 0.135, n.s.]. These results indicate that the cognitive load task prevented the escalation of attentional bias toward complex alcohol stimuli, an effect independent of main effects of group or time [*F*_(1, 42)_ = 0.247, n.s.; *F*_(1, 42)_ = 0.233, n.s.] (see Figure 4). A significant Group × Time interaction emerged for complex alcohol stimuli but not for simple alcohol stimuli.

**Table 2:** Mean (SD) of time to first fixation and total fixation duration for alcohol stimuli.

Measure	Load group (*n* = 23)	Load group (*n* = 23)	No load group (*n* = 22)	No load group (*n* = 22)	*F*	ηp^2^
	Pre-task	Post-task	Pre-task	Post-task		
**Time to first fixation (s)**						
Alcohol simple (s)	0.041 (0.117)	0.029 (0.015)	0.019 (0.015)	0.023 (0.012)	0.75	0.02
15.6-7.4,-1.3499pt Alcohol complex (s)	0.028 (0.018)	0.013 (0.010)	0.033 (0.024)	0.014 (0.013)	0.31	0.01
**Total fixation duration (s)**						
Alcohol simple (s)	7.667 (2.278)	7.615 (2.197)	8.606 (1.420)	8.352 (1.874)	0.55	0.01
Alcohol complex (s)	3.963 (0.894)	3.936 (0.934)	3.887 (0.503)	4.477 (1.130)	4.29^*^	0.09

Time to first fixation reflects early attention, and total fixation duration reflects late attention. Alcohol complex: Real-life scenes including alcohol. ηp^2^ = partial eta-squared. Post-hoc effect sizes (Cohen's *d*) are reported in the text. *p < 0.05

Taken together, the present results revealed a consistent dissociation across the three outcome domains. As hypothesized, depleting visuospatial working memory selectively reduced self-reported craving (AUQ) in the Load Group, while early, stimulus-driven orienting toward alcohol cues remained unaffected in both groups. The groups diverged at the late maintenance stage: whereas the No Load Group exhibited a progressive escalation of fixation toward alcohol cues from pre- to post-task, the Load Group showed no such increase. This suppression was confined to complex, socially embedded cues and did not extend to simple beverage images. The overall pattern indicates that visuospatial cognitive load left the automatic capture of attention by alcohol cues intact while specifically preventing the subsequent strengthening of attentional engagement, most clearly for the contextually rich cues. This pattern is consistent with the hypothesized role of visuospatial working memory in the maintenance, rather than the initial detection, of attention toward alcohol cues.

## 4 Discussion

The present study examined the effect of visuospatial cognitive load on alcohol craving and attentional bias in problematic drinkers. Results demonstrated that visuospatial cognitive load reduced alcohol craving, and that early and late attentional processes showed distinctly different patterns. The craving reduction observed in the Load Group, reflected in the significant pre- to post-task decrease in AUQ scores, is consistent with the core prediction of EI theory. According to this theory, craving does not arise from an alcohol-related intrusion itself. Instead, it arises from the subsequent elaboration of that intrusion into vivid, multisensory imagery within working memory. The more fully this imagery is constructed, the more intense and persistent the resulting urge becomes (Kavanagh et al., 2005; May et al., 2015). By demonstrating that depleting VSWM reduced self-reported craving, the present findings support the interpretation that engaging VSWM may have interfered with imagery elaboration, preventing transient intrusions from developing into sustained urge, rather than simply distracting participants from alcohol-related thoughts in general. A visuospatial task therefore draws on the same limited pool of VSWM resources required for imagery elaboration, thereby preventing craving from escalating.

Specifically, while initial orienting toward alcohol cues remained unaffected across groups, the escalation of late attentional maintenance toward complex alcohol cues was selectively suppressed in the Load Group. Initial orienting reflects an automatic, stimulus-driven response rooted in strong learned associations with alcohol, and as such is relatively resistant to top-down cognitive intervention (Manchery et al., 2017; Wiers et al., 2007). Late attentional maintenance depends on elaborative top-down processing that actively draws on VSWM resources, consistent with the premise that gaze maintenance and imagery elaboration share the same visuospatial substrate (Van der Stigchel and Hollingworth, 2018). This premise predicts that occupying VSWM through a concurrent task should correspondingly reduce the resources available to sustain gaze on alcohol cues. The total fixation duration findings corroborate this prediction, and this reduction was evident only for complex alcohol cues. Solitary beverage images carry sufficient perceptual salience to capture attention through bottom-up, stimulus-driven processes, without requiring the recruitment of episodic drinking memories or the construction of elaborative sensory imagery. Because this form of attentional capture does not draw on the top-down VSWM resources that the load manipulation targets, simple alcohol cues remained unaffected by the intervention. By contrast, socially embedded scenes activate a richer network of episodic drinking memories and therefore demand greater elaborative engagement, rendering them more vulnerable to VSWM depletion (Bollen et al., 2022). The absence of a corresponding effect for simple stimuli is theoretically consistent with this account, though because stimulus type was analyzed using separate models rather than a formal three-way Group × Time × Stimulus Type interaction, this pattern should be regarded as tentative pending direct statistical confirmation (Nieuwenhuis et al., 2011). Overall, this pattern is consistent with an account in which visuospatial cognitive load affects later, top-down elaborative processing rather than the automatic detection of alcohol cues. The eye-tracking findings therefore provide complementary objective evidence that the manipulation affected late attentional maintenance, a process theoretically associated with imagery elaboration. This differential pattern can also be interpreted within Tiffany's cognitive processing model of craving. From this perspective, the absence of an effect on initial orienting indicates that automatic, cue-initiated processing remained relatively intact, whereas the absence of an increase in late attentional maintenance in the Load Group suggests that the effortful, non-automatic processing recruited following initial cue activation was constrained by the visuospatial working-memory load. Both theories can therefore account for the present dissociation between initial and late attentional processes, though they differ in how they characterize the content of this later processing. Tiffany's model emphasizes controlled processing surrounding activated or interrupted action schemata, whereas EI theory emphasizes the elaboration of intrusive thoughts into sensory imagery. Given that the present manipulation directly occupied visuospatial working-memory resources, EI theory offers a more specific account of the observed reduction in craving and alteration in late attentional maintenance. Because imagery elaboration and action-schema activation were not directly assessed, however, the present findings cannot fully distinguish between these two mechanisms. An additional alternative explanation pertains to the structure of the No Load condition. Although post-task state anxiety was included as a covariate in the primary analyses, the relatively unstructured waiting interval may have afforded participants in the No Load Group greater opportunity to mentally reinstate, rehearse, or elaborate the previously presented alcohol-related images. By contrast, engagement in the CBTT may have constrained such processing during the intervention interval. This procedural difference should therefore be considered alongside the proposed VSWM-interference mechanism when interpreting the between-group divergence in late attentional maintenance.

The present finding contributes to a broader literature suggesting that occupying working memory resources around the time of alcohol cue engagement can reduce craving in problematic drinkers. The present findings differ from those reported by Kaag et al. (2018), who found that working-memory load reduced alcohol craving when administered before, but not after, alcohol-memory retrieval. However, the two studies targeted different cognitive processes and used substantially different retrieval procedures. They employed a prolonged, multimodal, and personalized alcohol-memory retrieval procedure followed by working-memory training to examine whether post-retrieval load would disrupt memory reconsolidation. In contrast, the present study administered the CBTT following brief passive exposure to alcohol-related images and assessed state craving shortly thereafter. From the perspective of EI theory, brief cue exposure can trigger intrusive alcohol-related thoughts, and their elaboration into sensory imagery may continue after the external cue is no longer present. Because this ongoing elaboration depends on limited visuospatial working-memory resources, a visuospatial task administered shortly after cue exposure could interfere with the construction or maintenance of craving-related imagery. Thus, the present effect is more consistent with the short-term disruption of an ongoing elaborative process before craving had fully developed than with the reconsolidation-based modification of an already reactivated alcohol memory. This interpretation remains tentative, as the time course of imagery elaboration was not directly assessed. These results also extend the work of Skorka-Brown et al. (2015), who demonstrated that briefly playing Tetris reduces self-reported craving for a range of substances in everyday life using an ecological momentary assessment design. The present study replicates this craving-reducing effect within a controlled laboratory paradigm targeting alcohol-specific cues, and isolates it to the visuospatial component of working memory through use of the Corsi block-tapping task. Whereas prior research relied solely on self-reported craving, this study corroborates this reduction with objective, eye-tracking-based evidence of reduced late attentional engagement with alcohol cues. This effect was demonstrated against a control condition matched for screen engagement, strengthening the causal interpretation that the observed reduction reflects VSWM-specific interference rather than general distraction.

These mechanistic findings carry preliminary clinical implications. Given that attentional maintenance, rather than initial orienting, is the component most strongly linked to craving and relapse risk (Field et al., 2004), the selective suppression of late attentional bias observed here represents a clinically meaningful intervention point. Unlike conventional craving interventions, which typically require a therapeutic setting, professional supervision, or pharmacological administration, the Corsi block-tapping task is brief, non-invasive, and can be self-administered without specialized training. This accessibility is particularly relevant given that the majority of problematic drinkers neither seek nor receive formal treatment (Lee et al., 2025). Visuospatial tasks delivered through smartphone applications have already been shown to reduce craving for a range of substances in naturalistic settings (Skorka-Brown et al., 2015), raising the possibility that a mobile-based adaptation of the present paradigm could similarly function as an accessible craving management tool. Such an approach could be considerably less resource-intensive than existing pharmacological or psychotherapeutic interventions, and could complement, rather than substitute for, formal treatment by providing an accessible adjunctive strategy for individuals who are not yet engaged in, or able to access, more intensive care. Because exaggerated salience attribution to reward cues reflects shared reward and inhibitory–control mechanisms across substance-related and non-substance-related addictive behaviors (Balconi, 2021; Zilverstand et al., 2018), a brief visuospatial load procedure that selectively dampens cue-driven attentional maintenance may have relevance beyond alcohol, offering a low-cost adjunct for disorders characterized by heightened cue salience.

However, several limitations warrant consideration. First, the fixed task level did not account for individual differences in VSWM capacity. Future research should examine whether adaptive loading calibrated to individual capacity produces stronger or more consistent effects. Second, the brief 1,000-ms stimulus presentation in the free-viewing task precluded fine-grained time-course analyses of approach-vs.-avoidance dynamics. Prior research varied stimulus duration across 200, 500, and 2,000 ms and found that attentional bias toward alcohol cues emerged only at the longer durations, with subjective craving most strongly associated with attentional bias at the 2,000-ms duration (Field et al., 2004). The 1,000-ms presentation used here fell within this range and was sufficient to detect a difference in total fixation duration, but it remains shorter than the duration at which subjective craving has been most strongly linked to attentional bias in prior work. This duration may therefore have been insufficient to capture more fine-grained dynamics, such as whether initial orienting transitions into sustained maintenance or avoidance over the course of a trial. Adopting longer stimulus durations would permit a more detailed analysis of how attentional bias toward alcohol cues unfolds over time. A related constraint concerns the 60-Hz sampling rate, which is well suited to the fixation-based, area-of-interest measures analyzed here but does not resolve the rapid saccadic movements involved in early orienting. The absence of a Group × Time interaction on time to first fixation should be interpreted as a null result specific to that measure, rather than as evidence that all early oculomotor processes were unaffected. Third, the absence of a parallel VAS effect likely reflects a psychometric difference, as the multi-item AUQ captures multiple facets of urge more closely tied to imagery-based processing, whereas the single-item VAS prompts a deliberate, conscious evaluation that may be less sensitive to subtle reductions in elaboration-driven craving (Diamantopoulos et al., 2012). Fourth, visual complexity was not independently quantified or equated across the alcohol-related and non-alcohol images. Although the stimuli were rated for their relevance to alcohol, valence, and arousal, and were analyzed primarily according to the simple-complex distinction, time to first fixation and total fixation duration can themselves be influenced by low-level image properties such as luminance, contrast, edge density, and image entropy (Parkhurst et al., 2002). These unmeasured differences may therefore have influenced the fixation measures reported here or the comparisons between simple and complex stimuli. Fifth, because imagery elaboration was not directly measured and the craving and attentional outcomes were analyzed separately rather than within a formal mediation framework, the present findings do not establish that changes in elaborative processing or late attentional maintenance mediated the reduction in craving. Sixth, a nominal baseline difference was observed in negative alcohol expectancy. Post-task STAI-S was included as a covariate because it differed significantly between groups and because previous research indicates that stress-related affective states are associated with alcohol cue-reactivity and attentional bias (Garland et al., 2012). AES-Negative was not included in the primary analyses and was not subsequently added on the basis of the statistical significance of the baseline group comparison alone, as the selection of covariates based on baseline significance testing is not recommended (Senn, 1994). The possibility that this baseline imbalance influenced the findings cannot be excluded and should therefore be considered in the interpretation of the results. Seventh, a sensitivity power analysis (G^*^Power 3.1.9.7; Faul et al., 2009) indicated that the achieved sample size (*N* = 45) provided 80% power to detect an effect of η_p_^2^ ≈ 0.044 for the primary within-subject Group × Time interaction. The two interaction effects central to the study's hypotheses, the Group × Time interaction for self-reported craving (AUQ; η_p_^2^ = 0.114) and that for total fixation duration to complex alcohol stimuli (η_p_^2^ = 0.093), both exceeded this minimum detectable effect. The sample size and these effect sizes also fall within the range reported by comparable studies using visuospatial-loading paradigms within the EI theory framework (*N* = 40–80; η_p_^2^ = 0.07–0.21; Andrade et al., 2012; May et al., 2010; Skorka-Brown et al., 2014). Power to detect small effects was nonetheless more limited for the secondary measures that yielded null results (alcohol VAS and early attentional bias), and these findings should be interpreted with corresponding caution. Finally, the sample was drawn from a single university, limiting generalizability to clinical or culturally distinct populations.

Future studies should employ larger and more diverse samples to enhance statistical power and improve the external validity of findings, while ensuring baseline equivalence in alcohol expectancy through stratified randomization. Additionally, the absence of a cue-exposure paradigm may have constrained baseline craving levels. Given that the present effects were observed under relatively low baseline craving, it remains unclear whether visuospatial cognitive load exerts comparable reductions when craving is elevated. Future studies should therefore incorporate cue-exposure induction procedures to examine whether the intervention effect generalizes to higher craving states. Taken together, these limitations call for replication using adaptive VSWM loading, longer stimulus exposures, objectively quantified and equated visual stimulus properties, adequately powered designs with ecological momentary assessment, more diverse samples that include clinical populations, and designs that directly assess imagery vividness and action-schema activation alongside a non-visuospatial cognitive-load condition. In conclusion, the present study provides preliminary evidence that visuospatial cognitive load reduces alcohol craving and prevents the escalation of late attentional bias to complex alcohol cues in problematic drinkers, without affecting automatic initial orienting, thereby isolating the elaboration stage as a cognitively accessible locus for future craving management strategies.

## Data Availability

The raw data supporting the conclusions of this article will be made available by the authors, without undue reservation, subject to the scope of the participants' informed consent and the approval of the Institutional Review Board of Chung-Ang University. Requests to access the datasets should be directed to the corresponding author.
